# Metabolism of Enantiomers of Rhododendrol in Human Skin Homogenate

**DOI:** 10.3390/metabo12050412

**Published:** 2022-05-03

**Authors:** Lihao Gu, Kazuhisa Maeda

**Affiliations:** 1 Bionics Program, Tokyo University of Technology Graduate School, 1404-1 Katakura-machi, Hachioji City 192-0982, Tokyo, Japan; d1117001d4@edu.teu.ac.jp; 2 School of Bioscience and Biotechnology, Tokyo University of Technology, 1404-1 Katakura-machi, Hachioji City 192-0982, Tokyo, Japan

**Keywords:** alcohol dehydrogenase, enantiomers, chemical leukoderma, metabolites, raspberry ketone, rhododendrol

## Abstract

We reported that raspberry ketone (RK) is produced from rhododendrol (RD) in excised mouse skin. We confirmed that RK is also produced from RD in human skin homogenates. We also observed more conversion of RD to RK when the oxidized form of nicotinamide adenine dinucleotide (NAD^+^), a coenzyme of alcohol dehydrogenase (ADH), was added to human skin homogenates. Chiral column analysis of the consumption of RD enantiomers in human skin homogenates also showed that more of the R enantiomers of RD remained than the S enantiomers of RD. This suggests that the S-enantiomer of RD is more easily oxidized in human skin. We confirmed that RD is partially metabolized to RK in human skin, thus suggesting that ADH in the skin may be the main cause of the appearance of this oxidation product.

## 1. Introduction

In Asian countries, “white skin” has long been considered attractive and sought after by women. For this reason, many Japanese cosmetics companies continually research new whitening mechanisms and the development of new materials. However, a cosmetic product containing 2% 4-(p-hydroxyphenyl)-2-butanol (rhododendrol; RD), an active ingredient developed in 2008 and approved by the Ministry of Health, Labor, and Welfare under Japan’s Pharmaceutical Affairs Law, was recalled in 2013 for causing leukoderma after having been sold on the Japanese market for 5 years [1].

RD is a naturally occurring substance with two enantiomers, (R)-RD and (S)-RD. The two enantiomers were found to be present in *Rhododendron chrysanthum* [2] and *Rhododendron maximum* L. [3]. Leukoderma due to RD is a type of chemical leukoderma that is medically defined as acquired depigmentation caused by repeated exposure to certain agents that damage melanocytes [4]. The first case of chemical leukoderma was reported in the literature in 1939 by Oliver et al. [5] As a raw material for the production of RD, 4-(p-hydroxyphenyl)-2-butanone (raspberry ketone; RK) has also been reported to pose a risk of chemical leukoderma [6,7]. The mechanism of RK-induced chemical leukoderma is thought to be the oxidation of RK to quinone compounds in the presence of tyrosinase in melanocytes [8]; this oxidation releases large amounts of reactive oxygen species (ROS) [7,9]. RD-induced chemical leukoderma also involves the same mechanism as RK-induced chemical leukoderma, with the generation of ROS in the presence of tyrosinase and cytotoxicity of ROS in melanocytes [9]. In addition, other 4-position substituted phenol-induced chemical leukoderma follows the same mechanism [10]. Our previous study [11] identified the hydroxyl radical (∙OH) as the ROS responsible for melanocyte cytotoxicity.

We additionally found that a portion of RD is metabolized to RK in mouse skin and that alcohol dehydrogenase (ADH) may be associated with this metabolism [12]. ADH is an enzyme widely present in living organisms that plays an important role in the biotransformation of alcohol in the presence of its cofactor, the oxidized form of nicotinamide adenine dinucleotide (NAD^+^). Five types of ADH have been found in humans [13,14,15,16], three of which are present in the skin, mainly in the epidermis [17]. Regarding the oxidative stability of the R- and S-enantiomers of secondary alcohols, the S-enantiomer is more easily oxidized, whereas the R-enantiomer is more stable against oxidation reactions [18]. Ito et al. reported that human tyrosinase oxidizes both R- and S-enantiomers of RD and the oxidation rate of (S)-RD is approximately 1.5 times faster than that of (R)-RD [19], thus suggesting that (S)-RD is more susceptible to oxidation.

**Figure 1:**
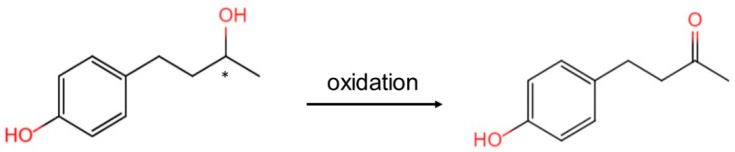
Oxidation of rhododendrol (RD) to raspberry ketone (RK). *: asymmetric carbon.

Therefore, the objective of this study was to determine whether RD is converted to RK (Figure 1) in human skin on the basis of an analysis of skin homogenates and to determine the oxidation ratio of the two enantiomers of RD using a chiral column.

## 2. Results

**Figure 2:**
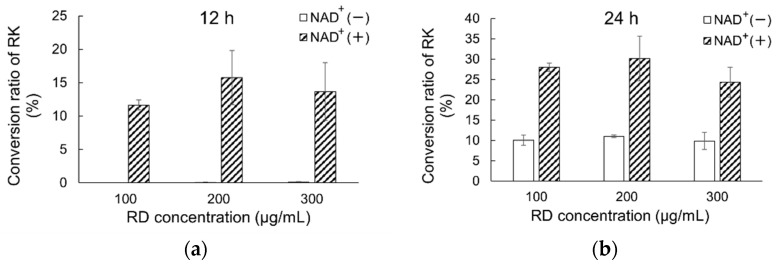
Comparison of metabolism of RD to RK in human skin homogenates exposed to different RD concentrations in the presence or absence of NAD^+^ ((**a**): after 12 h; (**b**): after 24 h). RK conversion rates after application of different RD doses (final concentration: 100, 200, or 300 μg/mL) in the presence or absence of NAD^+^. Data are presented as means ± standard deviation (n = 3). Concentrations of RK were converted to percentages for clarity; RD, rhododendrol; RK, raspberry ketone.

The metabolism of RD at different concentrations in human skin homogenates (0.8 mg protein/mL) was examined to assess the actual metabolism of RD in the skin after single and multiple applications of products containing 2% RD. The mass concentrations of RD and its metabolite RK in skin homogenates were determined over time (Figure 2). The overall recovery was 98.2 ± 3.8%. No change in RD concentration was observed in the control solution, but RD degradation was observed in skin homogenates containing high, moderate, or low concentrations of RD after 12 and 24 h. The percentage of RD consumed was found to be independent of concentration. Higher concentrations of RK were detected in the NAD^+^-containing group than in the group not containing NAD^+^.

The consumption of RD enantiomers was also examined at different concentrations in the presence or absence of 100 μmol/L NAD^+^ (Table 1). The results indicated that (S)-RD was more depleted than (R)-RD in the presence or absence of NAD^+^. The consumption ratios of (S)-RD to (R)-RD after 24 h in skin homogenates with high, moderate, and low concentrations of RD were (a) 1.42, 1.63, and 1.72 and (b) 1.10, 1.88, and 1.23.

## 3. Discussion

In our previous study [12], we confirmed that RD is oxidized to RK in mouse skin and reported the involvement of ADH in the skin in this process. We hypothesized that RD may be metabolized to RK in human skin, and the S-enantiomer of RD may be more easily oxidized to RK than the R-enantiomer. In this study, we confirmed that RD was oxidized to RK after 24 h in human skin homogenates The higher NAD^+^ concentration indicated that RD was metabolized to RK. These results suggested that RD oxidation occurred in the skin and that the addition of NAD^+^ promoted RD oxidation.

NAD^+^ has been reported to decrease with age [20,21], and the NAD^+^ levels in the skin of young adults (30–50 years) are more than twice those in middle-aged adults (51–70 years) [21]. Furthermore, ADH activity has been reported to be lower in alcoholics/older people than in the general population/young adults [22]. Therefore, RD might be less likely to be metabolized to RK in alcoholics and older people. However, the findings of this study are restricted to abdominal skin, which was taken from a 30-year-old female donor. The skin of alcoholics or elderly with low levels of ADH was not analyzed. Although we have proposed corresponding hypotheses, these speculations were not confirmed in this study.

Furthermore, the R- and S-enantiomers of RD are substrates for tyrosinase. A previous study [19] has indicated that the oxidation rate of (S)-RD by human tyrosinase to quinone derivatives is approximately 1.5-fold higher than that of (R)-RD. In the present study, we also observed that the oxidation rate of (S)-RD was higher than that of (R)-RD, with a ratio of 1 to 2.

The RD in RD-formulated cosmetics that caused chemical leukoderma was produced by reduction of RK with Raney nickel under a hydrogen atmosphere, and the R- and S-enantiomers were not separated [23], as they contained nearly equal amounts of (R)- and (S)-RD. We previously reported that both RD and RK generate ∙OH by tyrosinase, which leads to melanosome destruction and melanocyte cytotoxicity in the presence of tyrosinase [11]. We also reported that ∙OH generation capability, melanosome destruction, and cytotoxicity are stronger in RD than in RK at the same concentration [11]. In a previous study [12] and this study, we confirmed that RD is oxidized to RK in mouse and human skin and that ADH with its coenzyme NAD^+^ in the skin appears to be involved in this process. Because ADH was involved in the generation of RK from RD in the human epidermis in this experiment, it is likely that chemical leukoderma caused by RD-containing cosmetics is more likely to occur in people with lower ADH activity.

In conclusion, we confirmed that RD is partially metabolized to RK in mouse [12] and human skin, thus suggesting that ADH in the skin may be the main cause of the appearance of this oxidation product. Furthermore, Epidemiology Report 3, based on a nationwide survey of RD-induced leukoderma in Japan [24], indicated that most cases of RD-induced leukoderma occur in older people. Because RD has a stronger ∙OH generation capability, melanosome destruction, and melanocyte cytotoxicity than RK [11], the susceptibility of older people to leukoderma caused by RD is likely to be related to a decrease in ADH in the skin of older people.

## 4. Materials and Methods

### 4.1. Materials

RD was purchased from Wako Pure Chemical Industries, Ltd. (Osaka, Japan). RK was purchased from Sigma-Aldrich Corp. (St. Louis, MO, USA); NAD^+^ and ADH from yeast with a specific activity of 390 international enzyme units per milligram protein were purchased from Fujifilm Wako Pure Chemicals Co. (Osaka, Japan) Frozen human whole skin (155 cm^2^; abdominal skin from a 30-year-old female donor) was purchased from Biopredic International Ltd. (Saint Gregoire, France). BCA protein assay reagent was purchased from Thermo Fisher Scientific (Rockford, MA, USA). Other reagents were of analytical grade and were obtained from Wako Pure Chemical Industries, Ltd.

### 4.2. Methods

Human skin with most of the dermis scraped off was weighed and homogenized in PBS with a homogenizer to prepare 10% skin homogenate. The skin homogenate was centrifuged (3000× *g*) for 15 min, and the supernatant was collected and stored. The BCA protein assay reagent has been used to determine the protein concentrations of skin homogenates. RD (100, 200, or 300 μg/mL) alone or RD (100, 200, or 300 μg/mL) and NAD^+^ (100 μmol/L) were added to 5 mL of the supernatant and diluted to 10 mL with PBS. The solution was allowed to react under shaking at 32 °C and 250 rpm for 24 h, and 100 μL samples were collected after 2, 4, 8, 12, and 24 h. Subsequently, 10 μL of 60% perchloric acid was added to 90 μL of sample. The mixture was stirred, allowed to stand for 30 min, and centrifuged to remove the protein. The samples were stored at −30 °C for later analysis by high-performance liquid chromatography (HPLC). Standards of RD and RK were diluted separately and quantified using HPLC to establish calibration curves.

HPLC was performed with a Shimadzu HPLC system (Shimadzu Corporation, Kyoto, Japan), a Chiralcel OD-3R chiral column (4.6 × 100 mm; Daicel Corporation, Osaka, Japan), and an SPD-M20A photodiode array detector (Shimadzu Corporation, Kyoto, Japan) with a flow rate of 1.0 mL/min, injection volume of 10 μL, detection wavelength of 210 nm, and a mobile phase of 30:70 methanol/water.

## Figures and Tables

**Table 1:** Metabolism of RD enantiomers to RK in human skin homogenates exposed to different RD concentrations in the presence of NAD^+^ after 12 and 24 h.

	RD Concentration (μg/mL)	(S)-RD (%)	(R)-RD (%)	RK (%)	Consumption of (S)-RD (%)	Consumption of (R)-RD (%)	Consumption Ratio of (S)-RD to (R)-RD
12 h	100	50.24 ± 0.80	49.76 ± 0.80	-	-	-	-
	200	49.63 ± 0.85	50.33 ± 0.89	0.04 ± 0.07	-	-	-
	300	49.10 ± 0.60	50.83 ± 0.57	0.07 ± 0.08	-	-	-
12 h	100	43.51 ± 0.61	44.86 ± 0.78	11.63 ± 0.74	6.49	5.14	1.26
+NAD^+^	200	41.71 ± 2.31	42.55 ± 1.72	16.56 ± 3.77	8.29	7.45	1.11
	300	42.21 ± 2.44	44.13 ± 1.95	13.66 ± 4.31	7.78	5.87	1.32
24 h	100	44.11 ± 2.71	45.85 ± 3.77	10.04 ± 1.28	5.89	4.15	1.42
	200	43.15 ± 1.20	45.81 ± 1.40	11.04 ± 0.32	6.85	4.19	1.63
	300	43.78 ± 1.66	46.38 ± 0.84	9.85 ± 2.13	6.22	3.62	1.72
24 h	100	35.34 ± 1.76	36.64 ± 1.23	28.01 ± 1.04	14.66	13.36	1.10
+NAD^+^	200	30.31 ± 8.96	39.53 ± 3.65	30.16 ± 5.48	19.69	10.47	1.88
	300	35.41 ± 5.32	37.72 ± 5.84	26.87 ± 3.71	13.40	10.92	1.23

Note: the concentrations of (S)-RD, (R)-RD, and RK have been converted into percentages for clarity. Data are presented as means ± standard deviation (n = 3). RD, rhododendrol; (S)-RD, (S)-rhododendrol; (R)-RD, (R)-rhododendrol; RK, raspberry ketone; -, below the detection limit.

## Data Availability

Not applicable.
